# Amount of hepatic fat predicts cardiovascular risk independent of insulin resistance among Hispanic-American adolescents

**DOI:** 10.1186/s12944-015-0038-x

**Published:** 2015-05-01

**Authors:** Ran Jin, Ngoc-Anh Le, Rebecca Cleeton, Xiaoyan Sun, Jessica Cruz Muños, James Otvos, Miriam B Vos

**Affiliations:** Division of Pediatric Gastroenterology, Hepatology and Nutrition, School of Medicine, Emory University, Atlanta, GA 30322 USA; Biomarker Core Laboratory, Atlanta Veterans Affairs Medical Center, Decatur, GA USA; Department of Biostatistics and Bioinformatics, Emory University, Atlanta, GA USA; LipoScience Inc, Raleigh, NC USA; Children’s Healthcare of Atlanta, Atlanta, GA USA

**Keywords:** Non-alcoholic fatty liver disease, Hepatic steatosis, Lipids, Lipoproteins, Cardiovascular risk, Children and Adolescents

## Abstract

**Background:**

Nonalcoholic fatty liver disease (NAFLD) has emerged as the major pediatric chronic liver disease, and it is estimated to affect more than one third of obese children in the U.S. Cardiovascular complications are a leading cause of increased mortality in adults with NAFLD and many adolescents with NAFLD already manifest signs of subclinical atherosclerosis including increased carotid intima-media thickness.

**Methods:**

Volume of intrahepatic fat was assessed in 50 Hispanic-American, overweight adolescents, using Magnetic Resonance Spectroscopy. Lipoprotein compositions were measured using Nuclear Magnetic Resonance.

**Results:**

Plasma triglycerides (TG) (p = 0.003), TG/HDL ratio (p = 0.006), TG/apoB ratio (p = 0.011), large VLDL concentration (p = 0.019), VLDL particle size (p = 0.012), as well as small dense LDL concentration (p = 0.026) progressively increased across higher levels of hepatic fat severity, while large HDL concentration progressively declined (p = 0.043). This pattern of associations remained even after controlling for gender, BMI, visceral fat, and insulin resistance.

**Conclusions:**

Our findings suggest that increased hepatic fat is strongly associated with peripheral dyslipidemia and the amount of fat in the liver may influence cardiovascular risk. Further studies are needed to longitudinally monitor dyslipidemia in children with NAFLD and to examine whether the reduction of hepatic fat would attenuate their long-term CVD risk.

## Background

Nonalcoholic fatty liver disease (NAFLD) encompasses a wide spectrum of related disorders including simple steatosis, nonalcoholic steatohepatitis (NASH), and cirrhosis. It has become a common liver disease and affects approximately 10-30% of the general U.S. population [1]. Furthermore, with the rapid rise of childhood obesity [2], NAFLD is the most common cause of liver disease in pediatric populations. Hispanic-American children are frequently reported to have the highest prevalence of NAFLD even after controlling for the severity of obesity [3,4]. Individuals with NAFLD can develop end-stage liver disease; however, the global health risk of NAFLD is not confined to the liver. Cross-sectional studies in adults demonstrate a strong association of NAFLD with increased prevalence of clinical cardiovascular disease (CVD) [5,6]. Prospective studies show that CVD is a leading cause of death in adults with NAFLD, perhaps exceeding the risk from liver-related mortality [7,8]. Many adolescents with NAFLD already have subclinical atherosclerosis [9,10]. The growing evidence from these recent studies strongly emphasizes the importance of evaluating the CVD risk in pediatric NAFLD.

Lipids have been extensively investigated in NAFLD patients and the patterns associated with insulin resistance including high triglycerides (TG) and reduced high density lipoprotein (HDL) are commonly reported [11,12]. This alone could be responsible for the increased CVD associated with NAFLD because a high TG/HDL ratio is known to predict small dense low density lipoprotein (LDL) [13,14], a highly atherogenic particle [15,16]. However, while this pattern has been described in NAFLD, less is known about the relationship of lipoprotein particle size and number to the severity of NAFLD, especially in the pediatric population. In our current study, we aimed to evaluate the lipoprotein particle profile in a group of overweight, Hispanic-American adolescents who presented with a wide range of hepatic fat as measured by state-of-the-art magnetic resonance spectroscopy (MRS) methodology. This allowed us to isolate the effects of hepatic fat from body mass index (BMI) and insulin resistance which also may influence CVD risk. Here, we reported that the severity of hepatic fat has a strong association with a more atherogenic phenotype of lipoproteins.

## Results

Among these Hispanic-American adolescents (n = 50) who routinely had high consumption of sugar-sweetened beverages (SSBs), the prevalence of NAFLD (identified as hepatic fat > 5% by MRS) was 74% (n = 37). When stratified by the degree of intrahepatic fat, subjects with greater quantities of liver fat had increased alanine aminotransferase (ALT) (p < 0.001), visceral fat (p = 0.053), insulin (p = 0.008), and insulin resistance (indicated by HOMA-IR and adipo-IR, p = 0.019 and p = 0.002, respectively) (Table 1). The proportion of males was markedly increased in the category of the highest hepatic fat severity (p = 0.005) (Table 1).

**Table 1:** **Baseline characteristics of the study population stratified by hepatic fat (%)**

**Parameters, mean (SD)**	**Hepatic fat < 5%**	**Hepatic fat ≥ 5% and < 10%**	**Hepatic fat ≥ 10%**	**p-value**
**n**	**13**	**18**	**19**	
Age (years)	14.5 (1.85)	13.8 (2.23)	13.5 (2.91)	0.450
Male (n, %)	5 (38.5)	3 (16.7)	13 (68.4)	0.005
Weight (kg)	82.9 (19.8)	79.6 (17.4)	81.8 (13.9)	0.853
BMI z score	1.94 (0.26)	2.04 (0.32)	2.24 (0.43)	0.058
ALT (U/L)	16.9 (7.27)	19.8 (8.04)	73.6 (111)	<0.001
Hepatic fat (%)	3.95 (0.64)	7.25 (1.63)	15.6 (4.85)	<0.001
Visceral fat (cm^2^)	86.6 (35.7)	71.0 (23.3)	114 (48.8)	0.053
Glucose (mmol/L)	5.28 (0.89)	5.27 (0.71)	5.22 (1.08)	0.857
Insulin (mU/L)	17.3 (7.14)	29.3 (20.6)	41.4 (35.7)	0.008
HOMA-IR	3.94 (1.45)	7.03 (5.64)	10.2 (11.0)	0.019
Adipo-IR	13.4 (5.40)	23.4 (20.7)	63.7 (126)	0.002
hs-CRP (mg/L)	3.28 (3.47)	3.10 (2.27)	7.21 (7.95)	0.151

P-values were generated using ANOVA or alternatively Kruskal-Wallis tests for variables with a non-normal distribution. Sex was compared by the Fisher Exact test.

As presented in Table 2, plasma TG (p = 0.003), TG to HDL ratio (p = 0.006), TG to apolipoprotein (apo) B ratio (p = 0.011), large very low density lipoprotein (VLDL) concentration (p = 0.019), VLDL particle size (p = 0.012), as well as small dense LDL concentration (p = 0.026) progressively increased across higher levels of hepatic fat severity, while large HDL concentration progressively declined (p = 0.043).

**Table 2:** **Plasma lipid and lipoprotein profiles in the entire cohort stratified by hepatic fat (%)**

**Parameters, mean (SD)**	**Hepatic fat < 5%**	**Hepatic fat ≥ 5% and < 10%**	**Hepatic fat ≥ 10%**	**p-value**
*Standard lipid profile*				
Cholesterol (mg/dl)	155 (21.8)	156 (31.6)	180 (50.9)	0.116
**TG (mg/dl)**	**75.9 (26.7)**	**126 (71.8)**	**187 (117)**	**0.003**
LDL (mg/dl)	98.5 (27.6)	102 (23.9)	117 (45.1)	0.247
HDL (mg/dl)	46.5 (8.48)	41.3 (7.75)	44.3 (9.45)	0.147
nonHDL (mg/dl)	109 (25.5)	115 (26.9)	136 (50.9)	0.113
**TG/HDL ratio**	**1.76 (0.95)**	**3.16 (2.02)**	**4.23 (2.49)**	**0.006**
apoB (mg/dl)	61.0 (17.9)	65.5 (16.2)	75.6 (28.7)	0.299
**TG/apoB ratio**	**1.27 (0.37)**	**1.96 (0.94)**	**2.53 (1.65)**	**0.011**
FFA (mEq/L)	0.81 (0.27)	0.80 (0.26)	1.15 (0.72)	0.071
*Lipoprotein profile by NMR*				
VLDL & Chy particles (nmol/L)	44.5 (21.6)	57.7 (24.0)	56.8 (31.0)	0.202
**Large VLDL & Chy**	**1.32 (0.64)**	**5.01 (5.79)**	**6.46 (5.61)**	**0.019**
Medium VLDL	13.9 (10.6)	23.9 (21.6)	25.1 (18.8)	0.202
Small VLDL	29.3 (14.5)	28.7 (13.1)	25.3 (12.4)	0.633
LDL particles (nmol/L)	864 (279)	945 (211)	1136 (481)	0.084
IDL	80.0 (42.8)	73.6 (66.2)	77.9 (64.1)	0.954
Large LDL	343 (121)	320 (188)	358 (165)	0.777
**Small LDL**	**441 (295)**	**552 (267)**	**776 (445)**	**0.026**
HDL particles (μmol/L)	31.1 (4.37)	30.0 (3.06)	31.7 (5.25)	0.409
**Large HDL**	**5.69 (2.42)**	**4.20 (1.68)**	**4.11 (2.37)**	**0.043**
Medium HDL	9.57 (9.57)	10.7 (4.25)	10.3 (4.23)	0.667
Small HDL	15.8 (3.28)	15.1 (4.79)	17.3 (5.04)	0.743
**VLDL size (nm)**	**44.8 (4.73)**	**49.4 (8.12)**	**53.1 (7.94)**	**0.012**
LDL size (nm)	20.7 (0.58)	20.6 (0.57)	20.5 (0.50)	0.664
HDL size (nm)	9.20 (0.45)	9.01 (0.32)	8.94 (0.41)	0.178

P-values were generated using ANOVA or alternatively Kruskal-Wallis tests for variables with a non-normal distribution. Bold indicated statistical significance.

The relationship between hepatic fat and lipid and lipoprotein profile was further evaluated by adjusting potential confounders (Table 3). Consistently, plasma TG, TG to HDL ratio, TG to apoB ratio, large VLDL concentration, VLDL particle size, as well as small dense LDL remained positively associated with the degree of liver fat after adjusting for gender, BMI z-score, visceral fat, ALT, insulin, and insulin resistance indexes (both hepatic and adipose) (p ≤ 0.05 for all).

**Table 3:** **Relationship of the lipid and lipoprotein profiles with hepatic fat (%) as a continuous variable**

**Variable**	**Model 1**	**Model 2**
Standard Lipid Profile		
Cholesterol (mg/dl)	0.006 (−0.005 – 0.017)	0.007 (−0.008 – 0.022)
**TG (mg/dl)**	0.053 (0.028 – 0.079)**	0.043 (0.006 – 0.081)*
LDL (mg/dl)	0.004 (−0.011 – 0.020)	0.013 (−0.010 – 0.035)
HDL (mg/dl)	0.001 (−0.009 – 0.010)	−0.004 (−0.017 – 0.010)
nonHDL (mg/dl)	0.007 (−0.008 – 0.021)	0.010 (−0.011 – 0.031)
**TG/HDL ratio**	0.052 (0.025 – 0.080)**	0.047 (0.002 – 0.092)*
**TG/apoB ratio**	0.046 (0.025 – 0.067)**	0.032 (0.001 – 0.063)*
apoB (mg/dl)	0.007 (−0.008 – 0.023)	0.012 (−0.011 – 0.034)
Lipoprotein Compositions		
VLDL&Chy particles (nmol/L)	0.007 (−0.017 – 0.031)	0.006 (−0.031 – 0.043)
**Large VLDL & Chy**	0.069 (0.022 – 0.116)*	0.077 (0.011 – 0.043)*
LDL particles (nmol/L)	0.010 (−0.007 – 0.026)	0.015 (−0.010 – 0.040)
**Small LDL**	0.045 (0.007 – 0.083)*	0.059 (0.003 – 0.121)*
HDL particles (μmol/L)	0.002 (−0.005 – 0.009)	0.003 (−0.007 – 0.014)
Large HDL	−0.013 (−0.037 – 0.011)	−0.021 (−0.059 – 0.016)
**VLDL size (nm)**	0.008 (0.001 – 0.155)*	0.016 (0.002 – 0.022)*
LDL size (nm)	−0.001 (−0.002 – 0.001)	−0.001 (−0.003 – 0.001)
HDL size (nm)	−0.001 (−0.003 – 0.001)	−0.002 (−0.005 – 0.001)

Model 1: Unadjusted.

Model 2: adjusted for gender, BMI z-score, ALT, visceral fat, insulin, and insulin sensitivity.

Data are expressed as β coefficient of change per one-unit change in % hepatic fat (95% confidence interval). **p < 0.001; *p < 0.05; Data were log2 transformed. Bold indicated statistical significance.

Furthermore, hepatic fat appeared to be specifically associated with the small dense LDL subclass, irrespective of total LDL cholesterol, insulin resistance, and inflammatory status (p < 0.05) (Table 4).

**Table 4:** **Beta coefficients for the association between hepatic fat and %small dense LDL particles**

Linear regression covariates *	**β (SEE)**	**P value**
LDL	0.308 (0.157)	0.038
LDL, Insulin resistance	0.330 (0.173)	0.043
LDL, Inflammatory indicators	0.355 (0.163)	0.023
LDL, Insulin resistance, Inflammatory indicators	0.362 (0.181)	0.035
LDL, Insulin resistance, Inflammatory indicators,	0.340 (0.186)	0.053
Sex, BMI z-score		

*Insulin resistance included plasma insulin, HOMA-IR, and adipo-IR; inflammatory indicators were ALT, hs-CRP. Data were log2 transformed when appropriate.

## Discussion

In this cross-sectional study of overweight or obese Hispanic-American adolescents, a higher “dose” of fat in the liver was associated with a more atherogenic dyslipidemia characterized by increased plasma triglycerides, larger VLDL size, as well as greater small, dense LDL particles. Importantly, this dyslipidemic profile associated with intrahepatic fat was independent of multiple classical risk factors for CVD including obesity and adiposity, hyperinsulinemia, and insulin resistance. These findings suggest that hepatic fat is not only a marker of CVD risk, but also may be an important mediator in the pathogenesis of early atherosclerosis.

Several large natural history studies have shown that CVD is increased in adults with NAFLD [17-19]. Specifically, presence of NAFLD increases the risk of stroke and heart attack 2 fold over the risk from metabolic syndrome [17], suggesting that NAFLD pathophysiology contributes something above and beyond the contribution of insulin resistance and obesity. The exact mechanisms of the NAFLD and CVD risk are not known but recent work demonstrated that NAFLD is closely associated with the impairment of endothelial function and morphology as well as increased prevalence of carotid artery disease [19,20].

The liver plays a critical role in the biological process of VLDL assembly and secretion. Both hepatic steatosis [21,22] and insulin resistance [23] are known to be associated with increased large VLDL concentrations. Fabbrini et al. postulated that this alteration of VLDL particles in NAFLD could result from a dissociation in the regulation of the VLDL-TG and VLDL-apoB secretion rate, and they demonstrated increased TG content of newly secreted VLDL particles in NAFLD [24]. In the setting of insulin resistance, delivery of free fatty acid (FFA) to the liver is augmented because of the failure to suppress lipolysis in the adipose tissue [25]. De novo lipogenesis (DNL) is upregulated in NAFLD [26] and is further stimulated by carbohydrate intake such as sugar sweetened beverages [27]. The combination of increased FFA flux to the liver plus increased DNL could explain the production of large, TG rich VLDL that occurs in NAFLD and was demonstrated in this study.

The mechanisms linking large VLDL to small dense LDL are not completely understood but appear to be relevant to NAFLD CVD risk. Overproduction of TG rich lipoprotein (TRL) activates the enzyme cholesterol ester transfer protein (CETP) [28] leading to an increase in the exchange of cholesterol ester and triglyceride between TRL and LDL. This CETP-mediated interaction generates excellent substrates for further lipolysis and clearance by hepatic lipase, promoting the formation of small dense LDL. It is well established that small LDL has low affinity to LDL receptors and is more susceptible to oxidative modification, which contributes to atherogenicity [29,30]. It is also possible that large VLDL could be selectively retained in the arterial intima and therefore may share with LDL the potential for promoting atherosclerosis [31,32].

Given the near-universal interrelationship between NAFLD and insulin resistance, it is widely believed that hyperinsulinemia and insulin resistance are key metabolic defects resulting in increased accumulation of hepatocellular triglyceride. However, it is also possible that primary dysregulation of lipoprotein metabolism, including overproduction and impaired secretion of hepatic triglycerides, could contribute to hepatic steatosis independent of insulin resistance [33,34]. A recent multi-ethnic study of atherosclerosis in adults [22] indicated a strong correlation between NAFLD and the lipoprotein derangements irrespective of insulin resistance. Here, we furthered and expanded upon this finding in the pediatric cohort by demonstrating that the severity of liver fat in adolescents was associated with increased concentrations of large VLDL and small LDL after controlling for insulin resistance indexes. This current study is important because Hispanic-American children are one of the highest risk groups for NAFLD and because it shows the relationship between hepatic fat and lipoproteins is present at the earliest stages of disease. Further, much of the research on NAFLD and CVD has focused on inflammation, oxidative stress, insulin resistance and NASH [35-37]. Here, we observed a “dose” effect of hepatic fat on cardiovascular risk, primarily represented by an elevation of the atherogenic, small LDL subfraction. This finding raises the question: while the amount of fat in the liver does not have long term prognosis for progression to cirrhosis, could the fat volume be predictive of CVD and therefore could fat reduction be useful in decreasing CVD risk?

Large HDL particles are believed to be more anti-atherogenic [38,39]. The lower concentration of large HDL particles as seen in our participants who had increased severity of hepatic steatosis could be indicative of less “functional” HDL particles. HDL particles are formed when newly secreted apoA-I interacts with ATP-binding cassette A1 (ABCA1) on the surface of hepatocytes. This interaction promotes the incorporation of phospholipids and cholesterol into the nascent HDL particles, which are crucial for accepting excess cholesterol from peripheral tissues and lipid-laden macrophages [40,41]. This so-termed cholesterol efflux capacity of HDL allows for particle enlargement and plays a key role in preventing carotid intima-media thickness and CVD morbidity [42]. We also observed a blurred association between the degree of liver fat and the concentration of large HDL particles after multivariable adjustment for BMI, visceral adiposity, and insulin resistance. It is possible that liver fat *per se* might not have an independent influence on HDL composition, or at least the contribution from hepatic fat could be mitigated by any other factors such as visceral adiposity [43]. It is also possible that, in the setting of insulin resistance, hyperinsulinemia interrupts HDL biogenesis via promoting phosphorylation and degradation of ABCA1 [44]. Our findings underscore the complexity of HDL metabolism given its nature of heterogeneity and constant remodeling. Carefully designed kinetic studies will be needed in the future to accurately delineate the altered metabolism of HDL in pediatric NAFLD.

There are several strengths of our study. First, MRS methodology provides us a precise and noninvasive measure of hepatic fat. This quantitative assessment of liver fat has shown close correlation with biopsy (r = 0.9) and is superior to other traditional methods such as MRI and Dixon imaging technique [45]. Moreover, we treated our data as both categorical and continuous variables to reduce the potential error/bias introduced by grouping. By performing linear regression models, we are able to adjust underlying confounders and well characterize the relationship between liver fat and the lipoprotein profile.

Our study is also subject to some limitations. Our findings were generated from a relatively small size of a group of Hispanic children, and they may not be generalizable to other races/ethnic groups. Large-scaled analyses stratified by race/ethnicity will be needed to fully explore differences in the relationship between NAFLD and lipoproteins. Also, we were not able to evaluate NASH and CVD risk because our study participants did not undergo liver biopsy. Only a few of the participants had elevated ALT and this makes it more likely that most had steatosis without substantial inflammation; however it remains unknown. Finally, we cannot rule out the possibility that the fasting measures of insulin resistance did not completely capture the information of insulin sensitivity. Given the fact that there is no single measure that can adequately address insulin resistance, we included a series of direct and indirect measures of insulin resistance in our regression model, such as HOMA-IR, adipo-IR, BMI, and visceral adiposity.

## Conclusions

In conclusion, our findings demonstrated that among overweight or obese Hispanic-American adolescents, increased volume of hepatic fat was strongly associated with a more atherogenic lipid profile independent of insulin resistance, including increased concentration of large VLDL, greater size of VLDL particles, as well as elevated small dense LDL particles. This could explain early manifestation of signs of subclinical atherosclerosis in adolescents with NAFLD. Future studies are needed to assess if treatments that reduce hepatic fat will also decrease cardiovascular risk in NAFLD.

## Methods

### Subjects and study design

A total of 50 Hispanic-American children (overweight, BMI z-score ≥ 85th percentile, n = 4; and obese, BMI z-score ≥ 95th percentile, n = 46) were recruited from pediatric clinics at Emory Children’s Center and from nearby community centers through flyers and presentations at community events. Eligibility criteria included self-identification as Hispanic, ages 11–18 years; BMI ≥ 85th percentile for age and gender; and average self-reported consumption of at least 3 servings of SSBs per day. We studied Hispanic children because of their disproportionately high risk of hepatic steatosis [3,46]. High consumption of SSBs was designed as an inclusion criterion in order to increase the probability of recruiting subjects who were likely to have significant steatosis [46,47] but without having been previously diagnosed and treated. Exclusion criteria included pregnancy; previously known liver diseases; diabetes or fasting glucose > 126 mg/dl; renal insufficiency (creatinine > 2 mg/dl); any chronic diseases requiring daily medication; acute illness within the past 2 weeks prior to enrollment (defined by fever > 100.4 °F); and anti-oxidant supplement/therapy within the past 4 weeks before enrollment.

This was a cross-sectional analysis of an ethnically matched group of children at high risk of NAFLD. All participants underwent an anthropometric assessment and a MRS procedure for the determination of intrahepatic fat. Their blood samples were collected after fasting overnight (at least 12 hours) for the evaluation of lipids and the lipoprotein profile. The study protocol was approved by both Emory University and Children’s Healthcare of Atlanta institutional review boards and informed consent (parental consent obtained for subjects < 18 years) and assent (when applicable) were obtained for each subject prior to the study visit.

### Measurements of hepatic fat and visceral adiposity

Hepatic fat was assessed by MRS using our previously described methods [48]. Briefly, we used a rapid 15-second acquisition technique obtained during a single breath hold. The sequence is constructed from five concatenated echoes using a fixed set of echo times (TE) (12, 24, 36, 48, and 72 ms), with each echo having a repetition time (TR) = 3000 ms, voxel = 3 × 3 × 3 cm^3^, 1024 points, and 1200 Hz bandwidth. The acquisition was repeated three times for reproducibility. Data were exported off-line for automatic processing with in-house software (Matlab, Mathworks, Natick, MA). Water and lipid magnitude spectra were analyzed by determining the area under the curve corresponding to a user-defined frequency range surrounding the corresponding water/lipid peaks (water peak: 4.6 ppm; lipid peak: 1.3, 2.0 ppm). The integrated magnitude signals at each TE were fit to exponential T2 decay curves, whereby the equilibrium signal (M_0_) and the relaxation rate (R2 = 1/T2) were determined by least-squares approximation. Using M_0_ for water and lipid, the T2-corrected hepatic lipid fraction was calculated from: % Hepatic Lipid = M_0lipid_/(M_0lipid_ + M_0water_).

Visceral adiposity was calculated using ImageJ software (NIH, Bethesda, DM, USA). From a 3D gradient-echo acquisition with a 3-point Dixon reconstruction [49], a single, “fat-only” image was isolated at the L4 vertebral body. A signal threshold was set manually for each subject such that the subcutaneous fat was completely identified (>90% of maximum signal). This threshold was automatically extended to the visceral region, producing a binary mask of fat and non-fat regions. Manual segmentation was performed to separate subcutaneous and visceral regions by using the intra-abdominal muscle and perineum as boundary landmarks. The vertebra was not included in the segmented region. Visceral adiposity was calculated from the threshold volume of the segmented intra-abdominal region.

### Laboratory and lipoprotein analyses

Fasting blood samples were collected into EDTA-coated tubes and plasma was separated immediately. Plasma samples were protected from light and transported in ice pack to the Biomarker Core Laboratory for further processing (within 4 hours) using AU480 chemistry analyzer (Beckman Coulter, Inc.). Total cholesterol and TG were measured by enzymatic methods using reagents from Beckman (Beckman Diagnostics, Fullerton, CA). LDL cholesterol and HDL cholesterol were measured by homogeneous enzymatic assays (Sekisui Diagnostics, Exton, PA). Plasma levels of glucose, insulin, apoB, and high sensitivity C-reactive protein (hs-CRP) were determined using immunoturbidometric methods (Sekisui Diagnostics, Exton, PA). Liver enzyme ALT was measured by the Emory University Hospital clinical laboratory.

Lipoprotein particle concentrations and sizes were measured by NMR spectroscopy using a 400-MHz NMR clinical analyzer and the LP3 deconvolution algorithm (LipoScience Inc., Raleigh, NC) [50]. In brief, the NMR method uses the characteristic signals broadcast by lipoprotein subclasses of different size as the basis of their quantification. Each lipoprotein subclass signal emanates from the aggregate number of methyl groups on the lipids contained within the particle. This number is largely dependent on lipoprotein particle diameter and independent of lipid composition; thus, the amplitude of each lipoprotein subclass signal is directly proportional to the number of subclass particles emitting the signal. The subgroup of large VLDL and chylomicron particles were not able to be differentiated by this NMR technique, and in the current study, this subgroup was considered mainly as large VLDL because samples were obtained in a fasting state.

### Indexes of insulin resistance

Insulin sensitivity was assessed by the homeostasis model of assessment - insulin resistance (HOMA-IR) and the newly defined index adipo-IR [51]. HOMA-IR was calculated by glucose (mmol/L) × insulin (mU/L) / 22.5 at the fasting state, and adipo-IR was calculated by fasting FFA (mEq/L) × insulin (mU/L).

### Statistical analyses

We treated “hepatic fat” as both a categorical and a continuous variable. Demographic, anthropometric, and metabolic parameters of the study population were compared across stratifications of hepatic fat using ANOVA or alternatively Kruskal-Wallis tests for variables with a non-normal distribution. Sex was compared by the Fisher Exact test. Linear regression models (unadjusted and adjusted) were used to further assess the relationship between hepatic fat and two sets of lipid parameters: 1) a standard lipid profile, and 2) an advanced lipoprotein profile by NMR. When appropriate, log transformations were used to normalize outcome variables with skewed distributions. Statistical analysis was performed using SAS (version 9.3, Cary, NC).

## Abbreviations

ALT: Alanine aminotransferase
ABCA1: ATP-binding cassette A1
BMI: Body mass index
CVD: Cardiovascular disease
CETP: Cholesterol ester transfer protein
DNL: *de novo* lipogenesis
FFA: Free fatty acid
hs-CRP: High sensitivity C-reactive protein
HDL: High density lipoprotein
LDL: Low density lipoprotein
MRS: Magnetic resonance spectroscopy
NMR: Nuclear magnetic resonance
NAFLD: Nonalcoholic fatty liver disease
NASH: Nonalcoholic steatohepatitis
SSBs: Sugar-sweetened beverages
TG: Triglyceride
TRL: TG rich lipoprotein
VLDL: Very low density lipoprotein
